# Sonochemotherapy of breast adenocarcinoma: an experimental in vivo model

**DOI:** 10.1007/s40477-014-0120-7

**Published:** 2014-08-06

**Authors:** Bahram Yousefian, Seyed Mohammad Firoozabadi, Manijhe Mokhtari-Dizaji

**Affiliations:** 1 Rafsanjan University of Medical Sciences, Rafsanjan, Iran; 2 Department of Medical Physics, Faculty of Medical Sciences, Tarbiat Modares University, Tehran, Iran

**Keywords:** Sonochemotherapy, Bleomycin, Breast adenocarcinoma, In vivo

## Abstract

**Purpose:**

Because the cytotoxic potential of hydrophilic drugs like bleomycin (BLM) is restricted by its low membrane permeability, the application of low-intensity ultrasound (US) on growing tumor cells enhances intracellular delivery of BLM after intratumoral administration, thereby potentiating its cytotoxicity. In the present study, the in vivo cell membrane permeability enhancement with US (1 MHz, 2, 5, and 10 min, I_SPTA_ = 2 W/cm^2^) is compared with the murine model of breast adenocarcinoma in BALB/c mice.

**Methods:**

Tumor induction was performed through a homograft surgery procedure. Mice were anesthetized before putting them in sonication situations. Sonications were done in an aquarium. Seven groups of the tumor-bearing mice, each consisting of eight mice, were sonicated without or after intratumoral injection of 0.1 ml BLM at different exposure times. The tumor volume was evaluated to assess the growth process by use of a digital caliper.

**Results:**

The results show that the BLM control group has a significant difference with BLM plus 10 min US on day 2 (*p* < 0.05). There is a significant difference between 2- and 10-min sonication on days 8 and 10 also. The difference between the Only US group and the other groups except Sham US was significant too (*p* < 0.05). Significant differences were seen only between the BLM plus US groups with Sham US and Only US control groups.

**Conclusion:**

It has been concluded that for significant permeabilization of the cell membrane, sonication time for more than 10 min is required. Significant difference between the Only US and other groups indicates that US has a promoting effect on cell division procedure, in spite of the no-carcinogen effect of the US.

## Abstract (de)

**Scopo:**

Poiché il potenziale citotossico di farmaci idrofili come la bleomicina (BLM) è limitato dalla scarsa permeabilità della membrana cellulare, l’applicazione di ultrasuoni a bassa intensità (US) sulle cellule tumorali in crescita aumenta la diffusione intracellulare di BLM, dopo somministrazione intratumorale, potenziandone così la citotossicità. Nel presente studio, il miglioramento in vivo della permeabilità delle membrane trattate con gli US (1 MHz, 2, 5, and 10 min, I_SPTA_ = 2 W/cm^2^) è stato confrontato con il modello di adenocarcinoma mammario in topi BALB/c.

**Metodi:**

L’ induzione del tumore è stata effettuata attraverso una procedura di chirurgia omografa. I topi sono stati anestetizzati prima di essere sottoposti ad ecografia, effettuata in acqua. Sette gruppi di topi portatori di tumore, ciascuno composto da otto topi, dopo o in assenza di iniezione intratumorale di 0.1 ml BLM, sono stati sottoposti ad ultrasuoni con diversi tempi di esposizione. Il volume di crescita del tumore è stato valutato mediante l’uso di un calibro digitale.

**Risultati:**

I risultati mostrano che il gruppo di controllo BLM ha una differenza significativa con il gruppo BLM dopo 10 min US al secondo giorno (*p* < 0,05). Vi è una differenza significativa tra 2 e 10 minuti di esposizione agli ultrasuoni anche all’ottavo e decimo giorno. La differenza tra il gruppo sottoposto agli utrasuoni e gli altri gruppi è molto significativa (*p* < 0.05). Differenze significative sono state osservate solo tra i gruppi BLM sottoposti ad ultrasuoni, non sottoposti ad ultrasuoni e solo ad ultrasuoni.

**Conclusione:**

Si è concluso che per una significativa permeabilizzazione della membrana cellulare, questa debba essere sottoposta agli ultrasuoni per più di 10 minuti. Differenza significativa tra i soli gruppi statunitensi e di altri indica che gli Stati Uniti ha un effetto sulla promozione procedura di divisione cellulare, nonostante l’effetto no-cancerogeno degli Stati Uniti.

## Introduction

Cancer remains a leading cause of morbidity and mortality despite knowledge of its molecular basis, detection, and treatment. One of the most common cancers is breast adenocarcinoma. Many cancers evade the curative endeavors of conventional therapies like surgical resection, chemotherapy, and radiotherapy. Because of the involvement of a vital vein or nerve, many are inoperable, metastatic at first presentation, fail to respond to treatment, or following successful initial treatment may subsequently recur [1–4]. Because of the side effects of anesthesia, usually old patients are not candidates for surgery. Radiotherapy has abundant side effects, and X and γ radiations are carcinogen factors.

Chemotherapy also has very bad side effects. So usually, a combined treatment is used to improve the efficiency of each one of the treatments. Therefore, the development of alternative therapies for such cancers is clearly an imperative. One of the revolutionary treatment methods for cancer is the combination of physical modalities and routine treatments.

Physical modalities such as ultrasound (US) waves, electric field, etc., with different specifications have been used in this relation [5–9]. The combination of such physical modalities with routine treatment methods often leads to a reduction of the chemical drug dose and a decrease or total deletion of many side effects of these drugs and increases the efficiency of the treatment on the tumor and also the preservation of normal tissues [10].

One of the first physical modalities that were used was US waves. US waves with different intensities and frequencies have different biological effects. The effect of US on biological tissues depends on some exposure factors—such as frequency, intensity, power, exposure time, mode of irradiation… [9, 11, 12]. US has a unique advantage over other physical modalities that can penetrate through body tissues; it is focused on small areas and shows a completely targeted performance.

There are two major effects related to US waves: heat production and cavitation. The range of diagnostic US intensities is between 0 and 2 W/cm^2^, and the frequencies are from 1 to 20 MHz; this range is in the low-level intensity US.

Until now, no investigation shows the adverse biological effect of low-level intensity US, which is used routinely in clinics. In this range of US waves, transient cavitation process is improbable. However, the reflection of US waves from the opposite side wall of the aquarium may lead to the production of standing waves and subsequent transient cavitation. To avoid the occurrence of this phenomenon in the aquarium, using a US absorber, we opted to use the progressive wave mode [13–16]. While using US, there are two main categories of heat production: hyperthermia and high-intensity focused ultrasound (HIFU). HIFU needs intensities at the kW range, so it was not used in our trial. Temperature rising in low-level intensities is less than 10 °C, but it is sufficient for occurring biological effects [17].

Bleomycin prescription is one of the routine treatment protocols for many cancers [18]. Cell death due to bleomycin happens in one of two ways [19]. If only a few thousand bleomycin molecules are present in the cell, the cell is arrested in the G2–M phase, becomes enlarged, and polynuclei and micronuclei are observed [19, 20]. The cell then dies in a slow process lasting about three doubling times [20]. If, however, the cell contains several million bleomycin molecules, it will be killed within a few minutes through pseudoapoptosis, where bleomycin short circuits the apoptotic pathway by creating the characteristic DNA fragmentation. This is followed by cell shrinkage, membrane blebbing, and chromatin condensation [18, 20, 21].

Bleomycin is an extremely toxic agent once inside the cell [22], but this very high intrinsic cytotoxicity is restricted by the inability of bleomycin to freely diffuse through the plasma membrane [19, 20, 23]. Thus, it has been shown in vitro that less than 0.1 % of the bleomycin added to the extracellular medium becomes associated with the cells [24]. Therefore, it is an essential need to improve the diffusion of bleomycin through the plasma membrane to decrease the dose of the drug and subsequently the side effects on normal tissues.

In the present study, the in vivo cell membrane permeability enhancement with US at 2, 5, and 10 min exposure times by low-level intensity (I_SPTA_ = 2 W/cm^2^) was compared to the murine model of breast adenocarcinoma in BALB/c mice.

## Materials and methods

### Equipment setup

**Fig. 1:**
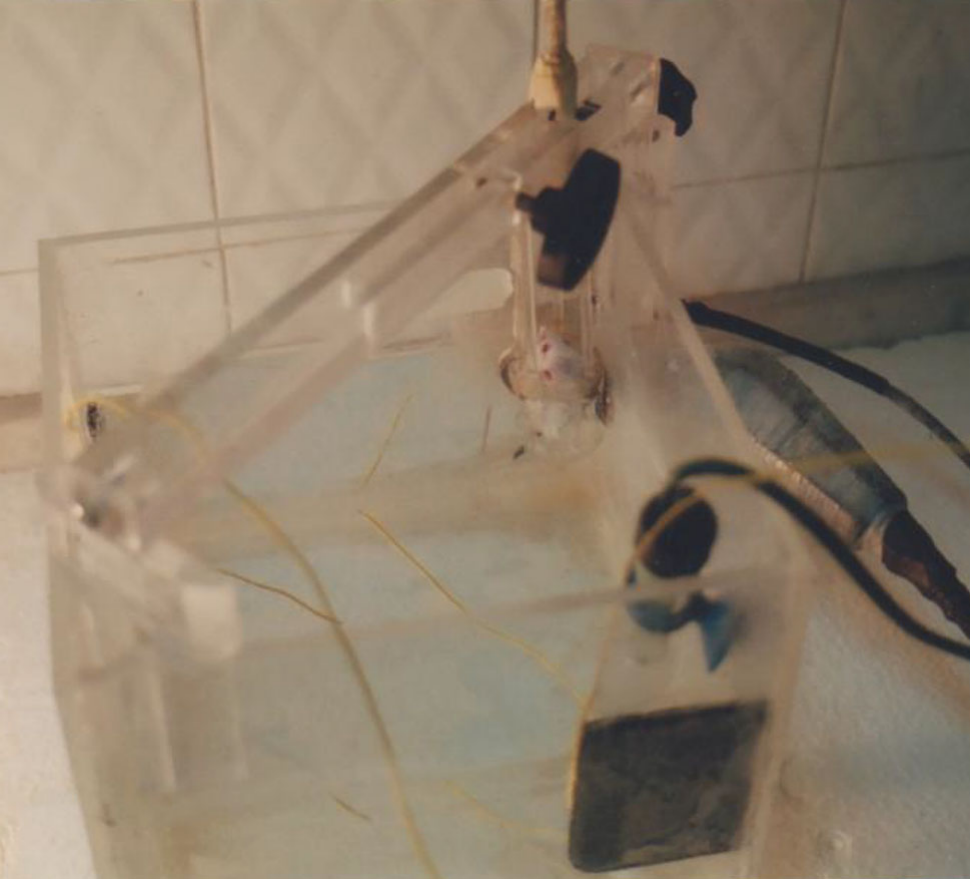
Mouse in the sonication setup

The photo of the experimental setup is shown in Fig. 1.

The US source was a 1 MHz US (Sonopulse 492, Enrof-Nonius Co., The Netherlands) with a PZT transducer probe (30-mm diameter and an area of 5 cm^2^) with pulsed mode and duty cycle of 80 %. Acoustic calibration for power and intensity of this device was carried out in degassed water using a US power meter balance (UPM-DT-10, Netech, Hicksville, NY, USA, ±1 mW). The intensity quoted was spatial average, temporal average (I_SATA_) of 2 W/cm^2^.

For exposure under controlled conditions, the apparatus used was a cubical tank with dimensions 25 × 25 × 20 cm^3^ constructed of Plexiglas and filled with degassed water. To eliminate the production of air bubbles between the probe and the tumor, a small amount of detergent was added to the tank. To perform the experiments under progressive wave conditions and to eliminate acoustic reflection and production of standing waves by the opposite wall of the tank, in front of the probe, a piece of an US absorber was attached to it.

The temperature of the water in the tank during tumor sonication was 35–38 °C. To control the temperature of the water in the tank, an aquarium heater equipped with a thermostatic control was used at all times, and the temperature was monitored during all experiments, with a digital thermometer. The probe held fixed in the tank through a circular hole at a distance from the corner of the tank makes it possible for the sonication of the mouse in the cage. The tumor is placed in the closest location to the probe surface.

The holder system for the tumor-bearing mouse was a cage constructed of fine polystyrene fiber suspended in the water in front of the probe. This system had the potential for movement in three directions and thus was completely adjustable for the manipulation of the tumor in front of the probe surface.

### Experimental procedure

Fifty-six healthy female BALB/c mice (6–8 weeks of age) were purchased from the Pasteur Institute (Tehran, Iran). They were kept at 22 °C with a natural day/night cycle for 10 days for adaptation. Spontaneous mouse mammary tumor (i.e., an invasive ductal carcinoma) was transplanted by implanting a 4-mm^3^ fragment into the right flank of the anesthetized mice through homograft surgery. Approximately 2 weeks after tumor transplantation, when the largest tumor diameter exceeded 5 mm (measured by a digital caliper), the animals were randomly divided into seven groups (eight animals for control groups and for each of the experimental groups).

### Drug preparation

One ml injectable saline was added to the bleomycin (BLM) vial, which contains 15 mg of crystalline powder BLM. This solution contained 25 U of drug. Thus, in each 0.1 ml of it, 2.5 U of BLM was present. Depending on tumor size, the appropriate dose of BLM was injected directly into the tumor. The injection was performed in two steps at two opposite points of the tumor. It was done for better drug spreading throughout the tumor volume.

For the anesthesia, we used a solution that contains 4 ml saline and 0.5 ml of etamine (10 %) (Alfasan Woerden, Netherlands) and 0.5 ml of xylazine (2 %) (Alfasan Woerden, Netherlands).

### Experimental groups

Seven experimental groups were present in this research.

- Bleomycin control group (BLM Cont.): In this group, only 0.1 ml of the BLM solution was injected into the tumor. No irradiation was used. The BLM content in the injected solution depends on the tumor size and was based on the standards in Table 1 [20].

**Table 1:** BLM content and dose-tumor volume dependence

Volume of tumor (mm^3^)	Drug dose (U^a^)	BLM content (mg/0.1 ml)
<100	0.50	0.30
Between 100 and 150	0.75	0.45
Between 150 and 200	1.00	0.61
Between 200 and 250	1.50	0.91
Between 250 and 300	2.00	1.21
>300	2.50	1.51

^a^One unit (*U*) contains 0.56–0.66 mg of BLM
- Sham control group (Sham Cont.): 0.1 ml of distilled water injected into the tumor.
- BLM plus 2-min US irradiation group: The mouse was anesthetized by an IP injection of the 0.01 ml anesthesia solution for each gram of its body weight. Then, 0.1 ml of the BLM solution was injected into the tumor. After 3 min, the tumor irradiated with pulsed US waves at 2 W/cm^2^, with 80 % duty factor for 2 min at the prepared setup.
- BLM plus 5-min US irradiation group: The mouse was anesthetized by an IP injection of the 0.01 ml anesthesia solution for each gram of its body weight. Then, 0.1 ml of the BLM solution was injected into the tumor, and after 3 min, the tumor irradiated with pulsed US waves at 2 W/cm^2^, with 80 % DF for 5 min at the prepared setup.
- BLM plus 10-min US irradiation group: The mouse was anesthetized by an IP injection of the 0.01 ml anesthesia solution for each gram of its body weight. Then, 0.1 ml of the BLM solution was injected into the tumor, and after 3 min, the tumor irradiated with US waves at 2 W/cm^2^, with 80 % DF for 10 min at the prepared setup.
- Sham US group: The mouse was anesthetized and put in the same situation as the irradiation groups at the setup, but no irradiation was applied to it. No BLM was used.
- Only US group: The mouse was anesthetized by an IP injection of the 0.01 ml anesthesia solution for each gram of its body weight; no BLM injection was used, and after 3 min, the tumor irradiated with pulsed US waves at 2 W/cm^2^, with 80 % DF for 5 min at the prepared setup.

The tumor diameters were measured every 48 h using a 0.02 mm digital caliper, and the tumor volume was calculated by standard formula. The formula most often used to measure the tumor volume is *V* = *ab* ^2^ *π*/6, in which a is the longest diameter and b is the next longest diameter perpendicular to a [21].

Using Microsoft Excel, the data were processed and the graph of tumor growth delineated and rendered. Analyzing the data was performed using SPSS 18 software.

## Results

**Fig. 2:**
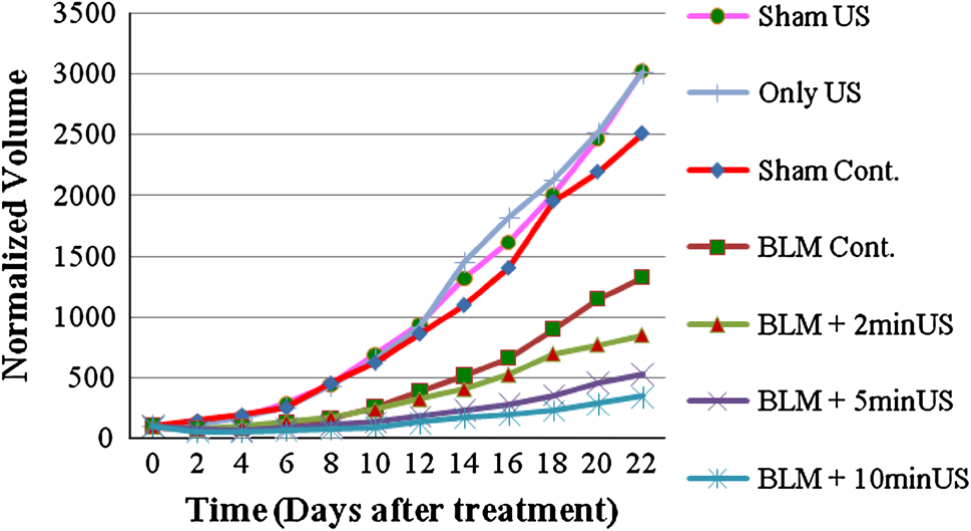
Tumor growth curve for seven groups of Balb/C mice (eight mice per every group) bearing tumors of murine breast adenocarcinoma:* filled circle* Sham US,* vertical line* Only US,* filled triangle* Sham Control,* filled square* BLM control,* filled triangle* BLM +2 min US,* multi symbol* BLM +5 min US,* asterisk* BLM +10 min US

The growth curve of the tumor in the experimental groups, between the treatment day and the 22nd day, is shown in Fig. 2.

Analyzing the data with SPSS showed that there is no difference between the experimental groups at treatment day (*p* < 0.05).

The growth of tumor in the Sham control group from day 2 until day 6 was significantly more than other groups except Sham US; nevertheless, tumor growth was significantly less than the Only US group. On days 8–14, its difference with the BLM plus 2-min US group became insignificant. During days 10–12, the growth of tumor in the Sham control was significantly more than the BLM plus 10-min US and less than the Only US group. During days 16–18, the growth of tumor in the Sham control was significantly more than the BLM plus 5- or 10-min US and less than the Only US group. On day 20, the growth of tumor in the Sham control was significantly more than the BLM plus 10-min US and less than the Sham US and Only US groups. On day 22, the growth of tumor in the Sham control was significantly more than the BLM plus 5- or 10-min US and less than the Sham US and Only US groups (*p* < 0.05, SEM = 364.83013).

The BLM control group on day 2 shows significant difference with all groups except the BLM plus 2- or 5-min US groups. On days 4, 6, and 8, the growth of tumor in the BLM control group was significantly more than all the groups except BLM plus US groups. On all other days, it does not have a significant difference compared with other groups except the Sham US and Only US groups, where its growth was less than them (*p* < 0.05, SEM = 270.906).

The growth of tumor in the BLM plus US-treated groups was significantly less than in the Sham groups and the Only US group on days 2, 4, and 6.

On days 8–10, the BLM plus 2-min US shows significantly more growth of tumor compared with the BLM plus 10-min US and less than the Sham US and Only US. In addition, the growth of tumor in the BLM plus 5- or 10-min US on day 8 was significantly less than the Sham Cont., Sham US, and Only US groups.

On day 10, the growth of tumor in the BLM plus 5-min US was significantly less than the Sham US and Only US, whereas the tumor volume in the BLM plus 10-min US was significantly less than the Sham control group and also the two mentioned control groups.

On day 12, the growth of tumor in the BLM plus 2- or 5-min US was significantly less than the Sham US and Only US groups, while the growth of tumor in the BLM plus 10-min US shows significant less volume compared with the Sham control and also the two other mentioned control groups.

On days 14 and 16, when the growth of tumor in the BLM plus 2-min US was significantly less than the Sham US and Only US groups, the growth of tumor in the BLM plus 5- or 10-min US was also less than in the Sham control group.

On day 18, the growth of tumor in the BLM plus 2-min US was significantly more than the BLM plus 10-min US and less than the Sham US and Only US groups. Other results are the same as on day 16.

The results on days 20 and 22 were the same as on days 14 and 16.

## Discussion

The effectiveness of chemotherapeutical drugs is dictated by the rate and extent the drug penetrates tissues and cells associated with the cancer, being limited by the side effects the drug exerts on tissues and cells not associated with the cancer. In this regard, the tumor blood vessel wall and the cancer cell membrane create physiological barriers for anticancer drugs.

The combination of US and chemotherapeutic drugs is currently exploited to enhance cancerous cells membrane permeabilization and uptake of the drug by the target cells [25–30]. Because US can be easily directed to specific sites or organs, it may be possible to increase the uptake of drugs and genetic material locally and selectively for the effective delivery of a drug into the cytosol [31].

The delivery of impermeable compounds like BLM by use of US into the cytosol has been demonstrated both in the in vitro [29, 30] and in vivo studies [32, 33]. The phenomenon of reversibly increasing the permeability of biological membranes is called sonoporation. The physical mechanisms by which this occurs have yet to be elucidated. However, mechanisms due to bubble disruption (formation of microjets and shock waves) and stable bubble oscillation (acoustic microstreaming) in ultrasonic fields are almost certainly implicated [34, 35]. This work investigates the permeabilization of murine breast adenocarcinoma cells to BLM in an in vivo experiment by use of US in three different exposure times.

The biophysical basis of the uptake of impermeable macromolecules, under our conditions, seems to be the formation of transient pores on the surface of the cell membrane [36].

There are two types of cavitation due to the use of US: transient cavitation and inertia cavitation [37]. The occurrence of transient cavitation has threshold exposure parameters that are higher than the utilizing exposure parameters in this trial [13–16]. Nevertheless, the occurrence of inertia cavitation is possible. Cell membrane permeabilization and enhancing the efficiency of the chemotherapy by use of low-level US by means of inertia cavitation is an established trial [7, 9]. Inertia cavitation has two separate effects:

- Microstreaming, which has the main role in cell membrane permeabilization.
- Heat production.

By reviewing the results, it can be understood that both of these phenomena play their roles in the trial.

SPSS results show that the growth of the tumor in the BLM plus US groups decreases compared with the Sham groups. However, the growth of the tumor in the Only US group increases compared with the other groups. This fact shows that BLM plus US reduces the volume of the tumor, but at the absence of BLM, the volume of the tumor will increase.

It can be because of the inertia cavitation action that enhances permeabilization of the cell membrane to BLM. Vice versa, in the absence of BLM, heat production leads to temperature enhancement, and in turn, temperature enhancement leads to vasodilatation and increased blood perfusion and better nutrition and oxygenation of the tumor cells. Thus, cell division exceeds and tumor volume increases faster compared to an unexposed tumor.

The insignificance of the difference of the BLM control group versus the BLM plus 2- and 5-min groups reveals the ineffectiveness of these US combination treatments. Nevertheless, the significant difference of the BLM control group vs. the BLM plus 10-min US group on day 2 shows a threshold time in this issue. It has been shown that US waves can unsettle the cell membrane structure [38, 39]. It also seems that low-level US is effective in making cell membrane permeabilized to BLM, but to become significant vs. the BLM control group on more days, the irradiation time must be longer than 10 min.

Nevertheless, both the BLM control group and the plus 10 min have a significant negative difference compared with the Sham US and Only US groups.

In this case, it seems the presence of physiological and psychological stresses in the Sham US and Only US groups and, as mentioned before, the US itself is a promoting factor and can lead to making significant negative differences for the BLM control group versus these two groups.

As shown in Fig. 2, after the eighth day, the graph begins to rise, and it seems the repair process of the pores is going to become complete. The return to the initial situation can gradually happen even longer.

The 10-min irradiation is outstanding in revealing treatment effects. Its difference with the BLM control group on the second day is significant at *p* = 0.045 (SEM = 10.32842). It seems that this treatment time is the most effective one to make turbulence in the cell membrane structure and establish more pores in the cell membrane. These events affect the membrane so efficiently, and therefore, the number of drug molecules that can enter the cell is more than other exposure times. To make a pore in the cell membrane, a completely defined deal of energy is required, so with increasing irradiation time, more energy will transfer to the cell membrane, and it seems we become closer to that defined energy. Nevertheless, there is a limit in making pores in the cell membrane. Beyond this limit, the pores will be irreversible, and the cell may erupt. It makes a perspective for tumor treatment without any drug and side effects by localized US.

However, it is expected that by increasing the exposure time beyond 10 min, the difference between the BLM control group and the BLM + X min becomes significant at *p* < 0.05 on more days.
